# Prediction of DNA N4-Methylcytosine Sites Based on a Position-Aligned Multi-branch Fusion Network

**DOI:** 10.34133/csbj.0147

**Published:** 2026-07-16

**Authors:** Hangyi Wang, Jian Li, Yaoping Ruan, Hailin Feng

**Affiliations:** School of Mathematics and Computer Science, Zhejiang A&F University, Hangzhou 311300, China.

## Abstract

While DNA N4-methylcytosine (4mC) plays regulatory roles in various organisms, its endogenous presence in mammalian genomes remains debated. Accurate identification of putative 4mC sites in eukaryotic genomes is impeded by scarce validated data and complex sequence dependencies. Although the mouse is a vital mammalian model for epigenetic studies, computational predictors capable of reliably screening murine 4mC candidate sites remain lacking. To address this, we propose TriAlignNet-4mC, a position-aligned triple-branch neural network designed for the precise and interpretable prediction of benchmark mouse 4mC candidate sites. Our framework unifies local biochemical properties, long-range contextual dependencies, and duplex-connectivity relations by integrating physicochemical descriptors, DNABERT embeddings, and duplex-connectivity-aware relational graph representations. Via position-aware alignment and late fusion, TriAlignNet-4mC generates comprehensive feature representations that markedly enhance prediction stability. Extensive benchmarking on a *Mus musculus* dataset demonstrates the model’s highly competitive performance. In independent testing, TriAlignNet-4mC achieved a sensitivity of 0.7937, a specificity of 0.8375, an accuracy of 0.8156, and a Matthews correlation coefficient of 0.6319, highlighting its robust generalization. Furthermore, ablation studies confirm the complementary contributions of the 3 branches: the contextual Transformer branch enhances sensitivity, while the graph-based structural branch improves specificity. Overall, rather than relying on exhaustive manual feature engineering, TriAlignNet-4mC is highly competitive with existing advanced predictors, exhibiting a distinct and robust trade-off between sensitivity and specificity.

## Introduction

DNA methylation is one of the most prevalent forms of epigenetic modification [1]. It regulates fundamental biological processes, including cell differentiation, embryonic development, and disease pathogenesis, by modulating gene expression without altering the DNA sequence [2]. Elucidating these regulatory mechanisms provides a foundation for interpreting the blueprint of life and identifies novel therapeutic targets for major diseases, such as cancer [3] and neurological disorders [4,5]. The most extensively characterized type, 5-methylcytosine (5mC), is formed by DNA methyltransferases at the C5 position of cytosine and typically functions to repress gene transcription [6]. In contrast, N4-methylcytosine (4mC) modulates gene expression by altering the physicochemical properties of DNA, thereby influencing transcription factor binding and chromatin architecture. It also contributes to genomic stability through DNA replication and repair processes [7]. Historically considered primarily a bacterial modification, recent discoveries have confirmed the existence of bona fide eukaryotic 4mC systems in specific nonmammalian lineages [8]. For example, studies in bdelloid rotifers demonstrate that bacterial-type 4mC can function as a verified eukaryotic epigenetic mark, supported by the biochemical and structural characterization of the corresponding DNA cytosine-N4 methyltransferase [9]. In contrast to these clear positive examples, the endogenous presence of 4mC in mammalian genomes remains highly controversial. While 5mC predominantly occurs within CpG islands to silence expression [1], early profiling via Single-Molecule Real-Time (SMRT) sequencing suggested a distinct regulatory landscape for putative 4mC marks in the *Mus musculus* genome [10]. However, the biological validity of endogenous mammalian 4mC remains highly debated. Quantitative liquid chromatography–mass spectrometry work reported no detectable N4-methyl-2′-deoxycytidine in mouse embryonic stem cells, brain, or liver [11]. Furthermore, subsequent technical studies concluded that SMRT sequencing can systematically overestimate 4mC when the modification is rare [12]. Therefore, computational frameworks designed for mouse datasets should be cautiously framed as tools for identifying putative 4mC candidate sites rather than definitive characterizations of endogenous mammalian biology. This functional divergence implies that these putative marks may possess distinct sequence motifs and structural determinants; consequently, computational frameworks optimized for 5mC [13] are ill-suited for 4mC identification [14]. Despite the growing recognition of its biological relevance, computational modeling of 4mC lags markedly behind that of 5mC [15]. Unlike the widespread silencing associated with 5mC, 4mC appears to regulate site-specific gene activation or repression, representing a potentially novel regulatory mode. Therefore, characterizing putative murine 4mC candidate sites computationally is essential for understanding whether this unique mechanism exists in specific spatiotemporal contexts. To the best of our knowledge, accurate computational predictors for mouse 4mC sites remain scarce. To address this gap, we developed a multi-branch deep learning framework for the precise screening of putative 4mC candidate sites. Extensive experiments on benchmark mouse data confirm the model’s robustness, facilitating an advanced computational understanding of potential 4mC patterns within the reference datasets.

DNA methylation detection methods fall into 2 primary categories: experimental “wet-lab” techniques and computational approaches. Wet-lab techniques [16], such as SMRT, identify modifications directly on the DNA strand by monitoring signal variations during polymerase synthesis. However, the widespread application of these methods is often hindered by high costs and limited throughput. Conversely, advancements in machine learning have facilitated the application of algorithms such as support vector machines, random forests, XGBoost, decision trees, and hidden Markov models to DNA methylation identification. The core of these methods relies fundamentally on handcrafted feature engineering [17]. Consequently, their predictive performance is strictly constrained by the quality of these pre-defined features. Furthermore, relying on fixed feature sets limits these models’ ability to capture long-range dependencies, complex contextual nuances, and underlying structural patterns [18,19]. Thus, traditional machine learning approaches often struggle to comprehensively decipher the deeper principles embedded in methylation patterns.

In contrast, deep learning methods have substantially improved methylation site prediction accuracy through end-to-end modeling of raw DNA sequences. Early research predominantly utilized convolutional neural networks (CNNs) to automatically extract local motifs, bypassing the need for laborious feature engineering. For instance, 4mCCNN [20] pioneered the application of deep learning to 4mC prediction, while MSNet-4mC [21] optimized this approach by employing multiscale receptive fields to efficiently capture local dependencies. Despite their proficiency with local features, CNNs inherently struggle to model global contextual information. To address this, recurrent neural networks were introduced to capture the long-range dependencies often overlooked by convolutional architectures [22]. For example, MultiScale-CNN-4mCPred [23] utilized bidirectional long short-term memory networks to process sequences bidirectionally, while 4mC-CGRU [24] adopted gated recurrent units to reduce computational overhead. Furthermore, 4mCMS [25] incorporated attention mechanisms to adaptively prioritize critical bases or regions within the sequence. However, despite these advancements—ranging from the local perception of CNNs and the sequential memory of recurrent neural networks to the weighted focus of attention mechanisms—these models share a fundamental bottleneck: they inherently treat DNA as a one-dimensional (1D) linear sequence, neglecting its higher-order spatial structure [26]. In parallel with these deep learning advancements, alternative machine learning paradigms, such as multifeature stacking frameworks [27] and domain-knowledge-driven ensemble learning [28], have also been explored for nucleic acid modification prediction; however, they similarly operate under the constraint of linear, 1D sequence representations.

Although recent deep learning approaches have yielded promising results, they remain constrained by 2 critical limitations. (a) Multidimensional information conflict: DNA sequences encode heterogeneous biological information, including local conserved motifs determining enzyme binding, long-range contextual dependencies, and structural features arising from the double-helix conformation. Existing models often indiscriminately blend these diverse features at the initial encoding stage. This early fusion frequently leads to feature conflict and information redundancy, thereby constraining the model’s expressive power. (b) Linear modeling constraints: Existing architectures remain strictly confined to a linear modeling perspective, treating DNA solely as a 1D sequence [26]. This reductionist approach fails to leverage the complex topological information inherent in the double-stranded structure. Consequently, these methods cannot effectively capture the higher-order interactions produced by the DNA double helix, which are critical for robust structural encoding.

To address the aforementioned challenges, this study proposes a triple-branch architecture predicated on a branch-independent encoding strategy. Our core design philosophy posits that distinct biological modalities must be represented by specialized encoding methods and processed by neural architectures tailored to their specific nature. This approach ensures the synergistic capture of multidimensional 4mC features without interference. (a) Branch-specific optimization: First, we standardize biochemical and physicochemical descriptors, linearly projecting them into a token-aligned representation. This method accommodates non-shift-invariant descriptors and enables stable, low-parameter fusion. Next, we pair Bidirectional Encoder Representations from Transformers (BERT)-based contextual embeddings with a Transformer network [29], leveraging self-attention to interpret sequence semantics and capture long-range dependencies. Finally, we model the constructed double-stranded DNA graph using a relation-aware graph neural network (GNN), which captures topology via explicit edge relations. This decoupled structure effectively resolves feature conflicts and information redundancy during the encoding stage. (b) Double-stranded graph encoding: We innovatively propose a double-stranded DNA graph encoding method. By treating nucleotides as graph nodes and defining edges based on the topological relationships within the double helix, this method transforms traditional linear sequences into graph structures rich in higher-order information. Consequently, this approach overcomes the dimensionality constraints of existing models, enabling the effective modeling of spatial DNA interactions. The core design of this architecture is driven by the complementary inductive biases of its branches. While the self-attention mechanism in the Transformer branch excels at treating DNA as a 1D sequence to capture global semantic dependencies, it lacks explicit constraints for local biophysical topologies. In contrast, the relational graph branch (G-StructRGCN) explicitly introduces topological priors—namely, main-strand continuity and cross-strand connectivity. This decoupling of “global semantics” and “local topology” overcomes the limitations of using a single sequence-based model for complex epigenetic environments.

The core contributions of this work can be summarized as follows:

• We designed a model based on a branch-independent encoding strategy. This architecture optimally pairs distinct biological modalities—biochemical features, contextual embeddings, and structural information—with specific network encoders (linear projection, Transformer, and relation-aware GNN). This design enables synergistic, interference-free integration, substantially enhancing the model’s expressive power.
• We proposed a duplex-connectivity graph encoding method to overcome the limitations of traditional 1D sequence models. By converting linear sequences into graph structures and integrating a relation-aware GNN (relational graph convolutional network [RGCN]) with explicit edge relations, this approach extracts cross-strand interactions and relational connectivity features that remain inaccessible to conventional sequence-based methods.
• We conducted extensive experiments on a benchmark mouse DNA methylation dataset. The results demonstrate superior predictive performance, confirming that our multi-branch architecture effectively captures complex methylation patterns and provides a robust tool for mouse epigenetic research.

## Materials and Methods

### Datasets

The dataset used in this study was obtained from the MethSMRT database [10], a repository of genome-wide methylation profiles derived from SMRT sequencing. To reduce redundancy and mitigate sequence bias, the CD-HIT [30] tool was employed with a similarity threshold of 0.7, removing sequences sharing greater than 70% identity. Subsequently, balanced datasets were constructed: the training set consists of 746 positive (4mC) and 746 negative (non-4mC) samples, while the independent test set contains 160 samples of each class.

All sequences were standardized to a fixed length of 41 bp, centered on the target cytosine. This window size adheres to established benchmark protocols, ensuring rigorous comparability with prior studies. Biologically, the ±20-bp flanking region provides sufficient context to capture the essential local sequence motifs and structural properties required for methyltransferase binding while minimizing irrelevant background noise from distal regions.

The positive 4mC labels in this murine dataset originate from the MethSMRT database, which identified modifications based on SMRT sequencing and polymerase kinetic signatures. We acknowledge that the reliability of identifying rare 4mC modifications in complex mammalian genomes using early SMRT platforms has been actively debated in subsequent studies. Consequently, this historical dataset is utilized here primarily as a standardized computational benchmark to ensure fair algorithmic comparisons with existing machine learning predictors. To further validate our model’s biological robustness and mitigate the potential noise in this specific benchmark, we additionally extended our evaluation to a large-scale, independent eukaryotic dataset (*Drosophila melanogaster*).

### Model construction

#### Overall framework of TriAlignNet-4mC

**Fig. 1.**
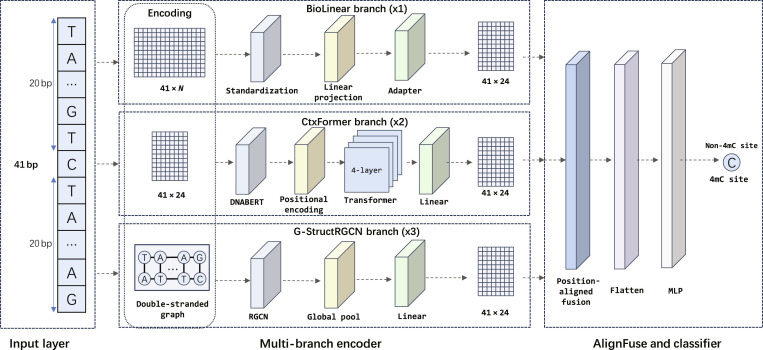
Schematic overview of the TriAlignNet-4mC model architecture.

As illustrated in Fig. 1, the proposed TriAlignNet-4mC framework comprises 3 primary modules: the input layer, the multi-branch encoder, and AlignFuse and classifier. (a) Input layer: Each DNA fragment is standardized to a fixed length of 41 bp, centered on the candidate cytosine. These sequences serve as parallel inputs for the subsequent branches, strictly preserving positional correspondence across different encoding modalities. (b) Multi-branch encoder. This module extracts multiview features from the categorical DNA input in parallel through 3 independent pipelines: the BioLinear branch for physicochemical characteristics, the CtxFormer branch for long-range contextual dependencies, and the G-StructRGCN branch for relational topology traits. Each branch adaptively processes its respective biological modality to output a dimensionally shape-aligned feature tensor of size ${\mathbb{R}}^{41\times 24}$, ensuring structural consistency prior to late fusion. All specific dimensionality reduction and feature extraction details are fully described in the “Multi-branch encoder” section. (c) AlignFuse and classifier. The outputs from the 3 branches are position aligned and concatenated into a unified representation. This fused feature tensor is flattened and processed by a multilayer perceptron classifier to predict whether the central cytosine is a 4mC or non-4mC site.

### Encoding

The encoding stage transforms raw DNA sequences into computational representations that capture their intrinsic biochemical, contextual, and structural characteristics. This process converts categorical nucleotide data into standardized numerical, physicochemical, and graph-based formats suitable for deep learning architectures. Specifically, 3 distinct encoding modalities are employed: biochemical feature encoding, contextual embedding, and structural graph encoding.

#### Biochemical feature encoding

The biochemical encoding strategy is designed to capture local sequence composition and physicochemical properties, which are critical for characterizing sequence motifs associated with 4mC modifications. To transform biological sequences into computational representations, we employ 2 complementary encoding schemes: numerical encoding and electron–ion interaction potential (EIIP) encoding [31].

##### Numerical encoding

DNA sequences comprise 4 nucleotides (A, G, C, and T), distinct in their base-pairing rules and molecular structures. To transform these categorical entities for computational processing, each nucleotide is mapped to a unique integer index. This encoding allows downstream models to treat DNA sequences analogously to text, facilitating the identification of short motifs based on index patterns. The mapping is defined as

$$\begin{array}{l} f_{\mathrm{num}}\left(A\right)=0\;f_{\mathrm{num}}\left(G\right)=1\\ f_{\mathrm{num}}\left(C\right)=2\;f_{\mathrm{num}}\left(T\right)=3 \end{array}$$ (1)

Thus, a DNA sequence $S=\left(s_{1},{\;s}_{2},\ldots ,{\;s}_{41}\right)$ yields a discrete vector of length 41.

##### EIIP encoding

Supplementing categorical identity, nucleotides possess intrinsic physicochemical properties that govern DNA stability and protein recognition. To integrate these features, we employed EIIP values, which quantify the electronic properties of each nucleotide and reflect its capacity for electron transfer. This mapping translates biological attributes into continuous values, enabling the model to leverage the physicochemical signals underlying DNA methylation. The mapping is defined as

$$\begin{array}{l} f_{\text{EIIP}}\left(A\right)=0.1260\;f_{\text{EIIP}}\left(G\right)=0.0806\\ f_{\text{EIIP}}\left(C\right)=0.1340\;f_{\text{EIIP}}\left(T\right)=0.1335 \end{array}$$ (2)

Consequently, each 41-bp sequence is transformed into a continuous vector of length 41, representing the physicochemical profile of the DNA fragment.

By integrating numerical and EIIP encodings with supplementary statistical and physicochemical descriptors, we construct a comprehensive biochemical feature set. These features are standardized and linearly projected into a token-aligned representation to facilitate downstream fusion. This projection strategy preserves positional consistency while mitigating overfitting and stabilizing the training process.

#### Contextual embedding

While local biochemical encodings capture immediate sequence composition, they lack the capacity to model the long-range semantic dependencies that govern biological function. To address this limitation, we employ DNABERT [32], a pre-trained Transformer model [29] specialized for genomic data. Each sequence is tokenized into overlapping 6-mers and mapped to a 768-dimensional (768D) embedding space.

To optimize computational efficiency while preserving semantic integrity, the high-dimensional embeddings (768D) are compressed to 24 channels via a deterministic channel grouping and mean pooling strategy. Subsequent standardization yields compact feature vectors for each nucleotide position. This dimensionality reduction retains the rich contextual semantics encoded by DNABERT while substantially lowering the computational overhead for downstream processing.

The contextual embedding of a sequence is expressed as

$$X_{\text{BERT}}=\left(E\left(s_{1}\right),\;E\left(s_{2}\right),\ldots ,E\left(s_{41}\right)\right)\;E\left(s_{i}\right)\in {\mathbb{R}}^{24}$$ (3)

where $E\left(\cdot \right)$ denotes the standardized, 24-channel DNABERT embedding function.

#### Structural graph encoding

Biologically, DNA methylation is governed not only by linear sequence but also by the local topological structure and physical flexibility of the double helix. Structural studies demonstrate that DNA methyltransferases employ a “base-flipping” mechanism to access the target cytosine, a process necessitating the distortion of the sugar–phosphate backbone and disruption of base-pairing interactions [33]. Consequently, properties of the complementary strand—such as base-pairing strength and stacking energy—dictate local helix stiffness and deformability, serving as key determinants for enzymatic recognition (often termed “DNA shape readout”) [34]. To capture the cross-strand structural dependencies and base-pairing context, we model each DNA fragment as a duplex-connectivity-aware relational graph. It is crucial to clarify that this approach yields a sequence-derived dual-strand connectivity graph rather than a direct biophysical model of 3-dimensional (3D) DNA shape parameters or experimentally validated conformational states. In this representation, every nucleotide from both the sense and complementary strands is modeled as an individual node, yielding 82 nodes for a 41-bp sequence fragment. To formalize the message-passing pathways across the duplex, edges are explicitly constructed across 3 distinct relation types: (a) main-strand backbone edges, which link adjacent nucleotides along the primary sequence; (b) complementary-strand backbone edges, which link adjacent nucleotides on the opposite strand; and (c) cross-strand hydrogen-bond edges, which strictly connect each sense nucleotide to its complementary base on the antiparallel strand. It is important to note that while the complementary text sequence can be logically inferred from the main strand, the topological message-passing pathways in a neural network differ fundamentally from 1D sequence attention. In our RGCN, the cross-strand relations are assigned independent, learnable weights. This provides the network with explicit “cross-strand shortcuts” to model spatial interactions, such as the base-flipping mechanism required by methyltransferases, which a purely 1D Transformer struggles to isolate from global sequence attention.

Based on this connectivity definition, we explicitly establish 3 graph variants to evaluate the contribution of specific structural connections in our downstream ablation studies: (a) full graph: incorporates all 3 edge types to enable complete duplex message passing; (b) no hydrogen bonds graph: retains all 82 nodes and intrastrand backbone edges but removes all cross-strand hydrogen-bond relations, isolating the effect of interstrand communication; and (c) single-strand graph: retains only the 41 primary sequence nodes and main-strand edges, functionally degrading the graph architecture to a 1D sequence representation. This multirelational design provides explicit “shortcuts” for the RGCN to aggregate contextual traits across strands, overcoming the search-space limitations of purely 1D sequence encodings.

Node attributes are initialized from the pre-trained DNABERT hidden states and reduced to 8 dimensions via principal component analysis (PCA). By embedding DNA as a relation-annotated graph, spatial—rather than purely sequential—dependencies are directly incorporated, enabling the RGCN to learn the structural signatures of methylation sites.

Formally, the structural representation is defined as

$$\begin{array}{c} G=\left(V,\;E,\;R\right),\;\left|V\right|=82,\\ E\subseteq V\times V,\;r:E\to R,\\ h_{v}\in {\mathbb{R}}^{8}\;\forall v\in V \end{array}$$ (4)

where $r\left(u,\;v\right)$ assigns an explicit relation type (e.g., backbone on a strand or base-pairing) to each edge $\left(u,\;v\right)$ and $h_{v}$ denotes the PCA-reduced embedding of node v.

### Multi-branch encoder

Following the encoding stage, 3 parallel neural branches independently process the feature representations, each emphasizing a complementary facet of the DNA sequence. This module comprises the BioLinear branch, the CtxFormer branch, and the G-StructRGCN branch.

#### BioLinear branch(x1)

We represent each 41-bp fragment using position-wise biochemical descriptors, mapping them to a token-aligned space via a parameter-efficient projection. Let $s_{i}$ denote the nucleotide at position *i*, and let $\phi \left(s_{i}\right)\in {\mathbb{R}}^{d_{0}}$ represent the aggregated vector collecting numerical, EIIP, and supplementary statistical and physicochemical descriptors. The stacked biochemical matrix is defined as

$$X_{\mathrm{bio}}={\left[\phi \left(s_{1}\right),\;\phi \left(s_{2}\right),\;\ldots ,\;\phi \left(s_{41}\right)\right]}^{⊤}\in {\mathbb{R}}^{41\times d_{0}}$$ (5)

To mitigate scale disparities and generate a token-aligned representation, we employ a unified standardization and linear projection step:

$$Z_{\mathrm{bio}}=\mathrm{Std}\left(X_{\mathrm{bio}}\right)P+1b^{⊤},\;P\in {\mathbb{R}}^{d_{0}\times d},\;b\in {\mathbb{R}}^{d}$$ (6)

where $\mathrm{Std}\left(\cdot \right)$ denotes feature-wise standardization derived from training set statistics and $1\in {\mathbb{R}}^{41}$ represents an all-ones column vector.

Subsequently, a lightweight trainable adapter processes $Z_{\mathrm{bio}}$ prior to the late-fusion stage. This streamlined pipeline maintains positional integrity, mitigates overfitting, and stabilizes optimization by avoiding the complexity of convolutional or recurrent architectures.

#### CtxFormer branch(x2)

This branch processes DNABERT contextual embeddings generated for each nucleotide position. The original 768D token vectors are compressed to 24 channels via group reduction and standardization, yielding a compact position-wise representation. A linear projection maps these embeddings to a shared 24-dimensional space, augmented by sinusoidal positional encodings to incorporate absolute spatial information within the 41-bp window. Subsequently, a stack of Transformer encoder layers captures long-range dependencies through multihead self-attention and position-wise feed-forward networks, strictly preserving sequence length and positional alignment. The final encoder output is projected via a linear layer to produce the contextual representation $Z_{\mathrm{ctx}}\in {\mathbb{R}}^{41\times 24}$.

Let $X_{\text{BERT}}\in {\mathbb{R}}^{41\times 24}$ denote the standardized, 24-channel DNABERT embeddings. The input to the encoder is defined as

$$H^{\left(0\right)}=\mathrm{PE}\left(X_{\text{BERT}}\right)$$ (7)

The mechanism employs single-head attention:

$$\text{Attn}\left(Q,\;K,\;V\right)=\text{softmax}\left(\frac{{\mathrm{QK}}^{⊤}}{\sqrt{d_{k}}}\right)V$$ (8)

and multihead composition:

$$\text{MHSA}\left(X\right)=\left[{\text{head}}_{1},\;\ldots ,\;{\text{head}}_{h}\right]W^{O}$$ (9)

The encoder output maintains a length of 41 and is linearly projected to $Z_{\mathrm{ctx}}\in {\mathbb{R}}^{41\times 24}$.

#### G-StructRGCN branch(x3)

To explicitly encode the duplex-connectivity relations, we construct a dual-strand graph for each 41-bp window, comprising 82 nucleotide nodes representing both strands. Edges are assigned explicit relation types to capture strand continuity and cross-strand base-pairing. Node attributes are initialized via DNABERT embeddings and compressed from 768 to 8 dimensions using PCA. This aggressive compression is intentionally designed to maintain a lightweight parameter space during multirelational message passing over 82 nodes, effectively preventing severe overfitting.

An RGCN updates node states through relation-specific transformations and self-loops. Importantly, by assigning independent, learnable weight matrices to different relations (e.g., cross-strand hydrogen bonds), this mechanism provides explicit topological shortcuts to model spatial interactions like base-flipping, which are fundamentally overlooked by purely 1D linear sequence models. For a graph $G=\left(V,\;E,\;R\right)$ with node states $h_{v}^{\left(k\right)}$ at layer *k*, the RGCN update rule is defined as

$$\begin{array}{c} h_{v}^{\left(k\right)}=\sigma \left(\sum_{r\in R}\sum_{\;u\in N_{r}\left(v\right)}\frac{1}{c_{v,r}}W_{r}^{\left(k\right)}h_{u}^{\left(k-1\right)}+W_{0}^{\left(k\right)}h_{v}^{\left(k-1\right)}\right) \end{array}$$ (10)

where $N_{r}\left(v\right)$ denotes the set of neighbors connected to node *v* via relation *r*, $c_{v,r}$ is a normalization constant, and $W_{r}^{\left(k\right)}$ and $W_{0}^{\left(k\right)}$ represent the trainable relation-specific and self-loop weights, respectively.

Formally, the RGCN updates the 8-dimensional initial states into 24-dimensional hidden states at the final layer *K*, denoted as $h_{v}^{\left(K\right)}\in {\mathbb{R}}^{24}$. Subsequently, a global readout function (global mean pooling) aggregates the final node states across the entire duplex graph into a holistic graph-level structural context vector $g\in {\mathbb{R}}^{24}$:

$$g=\frac{1}{\left|V\right|}\sum_{v\in V}h_{v}^{\left(K\right)}$$ (11)

To explicitly fuse this global topological prior with the position-wise sequences (41 × 24) of the parallel branches, we implement a linear projection matrix $W_{g}\in {\mathbb{R}}^{984\times 24}$ and a bias vector $b_{g}\in {\mathbb{R}}^{984}$. The vector g is projected and reshaped into a tensor dimensionally aligned with the sequence window:

$$Z_{g}=\text{reshape}\left(W_{g}\;g+b_{g}\right)\in {\mathbb{R}}^{41\times 24}$$ (12)

Although the explicit one-to-one biological nucleotide mapping is integrated during the global pooling step, this dimensionally shape-aligned reshaping mathematically ensures that holistic duplex-connectivity traits are seamlessly broadcasted across the sequence window. This allows the structural priors to be directly concatenated with the local positional features in the late-fusion stage.

### AlignFuse and classifier

**Fig. 2.**
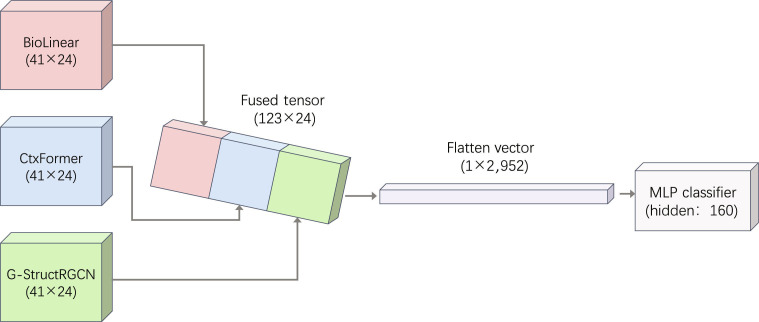
Schematic illustration of the position-aligned fusion mechanism.

The fusion strategy is critical for integrating the complementary information captured by the 3 parallel branches. As illustrated in Fig. 2, each branch outputs a strictly position-aligned feature matrix with identical dimensions of ${\mathbb{R}}^{41\times 24}$. To preserve the specific semantic contributions of each position without premature feature mixing, we employ a concatenation strategy along the token dimension (sequence length axis).

Specifically, the feature matrices $Z_{\mathrm{bio}}$, $Z_{\mathrm{ctx}}$, and $Z_{g}$ are concatenated to form a unified tensor $Z_{\text{fused}}\in {\mathbb{R}}^{123\times 24}$. This integration ensures that the biochemical, contextual, and structural features remain strictly aligned and preserved. The fused tensor is subsequently flattened into a vector $v\in {\mathbb{R}}^{1\times 2,952}$ (123 × 24 = 2,952) and fed into a 2-layer multilayer perceptron with 160 hidden units to compute the final prediction probability.

The mathematical formulation is defined as

$$\begin{array}{c} Z=\left[Z_{\mathrm{bio}};\;Z_{\mathrm{ctx}};\;Z_{g}\right]\in {\mathbb{R}}^{123\times 24}\\ \hat{y}=\sigma \left(W_{2}\;\phi \left(W_{1}\;\mathrm{vec}\left(Z\right)+b_{1}\right)+b_{2}\right) \end{array}$$ (13)

where “;” denotes concatenation along the token dimension, ϕ represents the nonlinear activation function, and $\mathrm{vec}\left(\cdot \right)$ denotes the flattening operation.

### Implementation details and regularization strategy

All models were implemented using the PyTorch 2.5.1 framework on an NVIDIA GeForce RTX 4060 Ti graphics processing unit, with an average training duration of approximately 1 min per fold. We utilized the pre-trained DNABERT-6 model as the backbone for context extraction. Training was conducted with a batch size of 128 for a maximum of 60 epochs.

To mitigate overfitting given the limited dataset size, we implemented a strict regularization protocol. First, the DNABERT model was frozen and utilized solely as a static feature extractor, preventing parameter updates during training. Consequently, the trainable components of TriAlignNet-4mC (comprising the projection layers, GNN, and Transformer encoder) total approximately 0.54 million parameters. This lightweight design substantially restricts the hypothesis space compared to end-to-end fine-tuning. Furthermore, dropout was applied at multiple stages—0.1 in the GNN module and 0.2 in the multi-branch fusion layer—to prevent neuronal coadaptation. Optimization was performed using Adam with an initial learning rate of 1 × 10^−3^ and a weight decay ($L_{2}$ penalty) of 1 × 10^−4^ to constrain weight magnitudes. To stabilize convergence, the learning rate was modulated via a cosine annealing warm restarts scheduler ($T_{0}=10$, $T_{\text{mult}}=2$, and $\eta_{\min}=1\times 10^{-5}$). Finally, an early-stopping mechanism was employed with a patience of 8 epochs, monitoring the validation balanced accuracy to halt training at the optimal generalization point. For model interpretation and motif discovery, the integrated gradients (IG) attribution was implemented using the Captum library. The attribution scores were computed relative to an all-zero baseline tensor in the compressed DNABERT embedding space, utilizing a path integral approximated via 50 Gauss–Legendre quadrature steps to ensure mathematical convergence and attribution accuracy.

### Performance evaluation

To objectively assess the predictive capability of the model, we employed both cross-validation and independent testing. Specifically, stratified 10-fold cross-validation was conducted on the training dataset to evaluate model stability and mitigate partition bias. The dataset was partitioned into 10 equal subsets; in each iteration, 9 folds served as the training set and 1 as the validation set, with the final performance reported as the average across all folds. Crucially, to entirely eliminate the risk of mathematical data leakage, all feature standardization (via StandardScaler) and graph node dimensionality reduction (via IncrementalPCA) were strictly fitted exclusively on the training folds during each cross-validation iteration. The validation and independent test partitions were transformed using only these pre-fitted historical parameters, ensuring an ironclad barrier against information leakage.

During cross-validation, the decision threshold was optimized independently for each fold. At each validation step, the threshold was swept across the range $\left[0,\;1\right]$, and the value maximizing balanced accuracy on the current validation fold was selected. For the final evaluation on the independent test set, we applied the specific threshold corresponding to the best-performing model checkpoint (i.e., the threshold tuned on its associated validation data). This protocol ensures that the decision boundary is determined solely by the validation distribution. Consequently, the independent test set—never seen during training—provides an unbiased estimation of the model’s generalization ability.

To quantitatively assess model performance, we employ 4 standard metrics: sensitivity (Sn), specificity (Sp), accuracy (Acc), and Matthews correlation coefficient (MCC). These are defined as follows:

$$\begin{array}{c} \mathrm{Sn}=\frac{\mathrm{TP}}{\mathrm{TP}+\mathrm{FN}}\\ \mathrm{Sp}=\frac{\mathrm{TN}}{\mathrm{TN}+\mathrm{FP}}\\ \mathrm{Acc}=\frac{\mathrm{TP}+\mathrm{TN}}{\mathrm{TP}+\mathrm{TN}+\mathrm{FP}+\mathrm{FN}}\\ \mathrm{MCC}=\frac{\mathrm{TP}\times \mathrm{TN}-\mathrm{FP}\times \mathrm{FN}}{\sqrt{\left(\mathrm{TP}+\mathrm{FP}\right)\left(\mathrm{TP}+\mathrm{FN}\right)\left(\mathrm{TN}+\mathrm{FP}\right)\left(\mathrm{TN}+\mathrm{FN}\right)}} \end{array}$$ (14)

where TP, TN, FP, and FN denote the number of true positives, true negatives, false positives, and false negatives, respectively.

Specifically, sensitivity quantifies the model’s capacity to correctly identify true methylated sites, while specificity measures the rejection rate of nonmethylated sites. Accuracy provides an overall measure of correctness; however, MCC—ranging from −1 to +1—is regarded as a more robust metric. By incorporating all 4 confusion matrix components, MCC offers a comprehensive evaluation of binary classification quality, particularly in distinguishing subtle decision boundaries.

## Results and Analysis

### Overall performance

To rigorously evaluate the predictive efficacy of TriAlignNet-4mC, we benchmarked it against 4 representative advanced predictors: the ensemble-based 4mCPred-EL [13], the feature-engineering-based i4mC-Mouse [35], the deep-learning-based 4mC-CGRU [24], and the recently proposed advanced deep learning framework iResNetDM [36]. Crucially, as 4mC-CGRU was originally optimized for the Rosaceae genome, we reimplemented its hybrid architecture and trained it from scratch using the identical mouse dataset splits to ensure a strictly fair architectural comparison.

Evaluations were conducted using both 10-fold cross-validation and independent testing to verify model robustness and generalization. In the cross-validation phase, the dataset was partitioned into 10 subsets (9 for training and 1 for validation), and performance metrics were averaged across the 10 iterations.

To strictly prevent any potential information leakage during the cross-validation and independent testing phases, all feature normalization and dimensionality reduction steps were strictly isolated within the training loop. Specifically, both the StandardScaler applied to the biochemical features and the IncrementalPCA utilized for compressing the DNABERT graph initializations were fitted exclusively on the training subset of each respective fold. The validation and independent test sets were subsequently transformed using only these pre-fitted parameters. This strict isolation protocol ensures that no distributional information or variance from the validation/test data could inadvertently influence the feature encoding phase, thereby guaranteeing the absolute integrity of our performance metrics.

In the 10-fold cross-validation analysis (Table 1), the proposed model demonstrated a highly competitive equilibrium between sensitivity and specificity compared to the baseline models. While the ensemble-based 4mCPred-EL attained competitive sensitivity, TriAlignNet-4mC substantially surpassed it in both specificity and MCC. This improvement suggests that the proposed multi-branch integration refines the decision boundary, effectively mitigating the high false-positive rates often inherent in ensemble voting schemes. As noted, i4mC-Mouse yielded a marginally higher MCC (0.6510 *vs* 0.6213), although TriAlignNet-4mC achieved a higher overall accuracy (0.8031 *vs* 0.7930). The competitive performance of i4mC-Mouse is likely attributable to its exhaustive handcrafted feature engineering. However, rather than relying on manual feature selection, TriAlignNet-4mC maintained a distinct and highly stable trade-off between sensitivity and specificity across folds, underscoring its automated robustness. Furthermore, the reimplemented 4mC-CGRU displayed competitive sensitivity but exhibited a marked deficit in specificity and MCC. This disparity indicates that standard deep learning architectures, when lacking explicit structural constraints, are prone to overpredicting positive sites, resulting in a bias toward high sensitivity at the cost of precision. Similarly, when evaluated against the advanced deep learning architecture iResNetDM, TriAlignNet-4mC proved highly effective. While iResNetDM provides a modern computational baseline, our model achieved higher overall accuracy and MCC, demonstrating that our decoupled multi-branch topology effectively extracts more discriminative representations than standard unified deep learning integrations.

**Table 1.** Performance comparison with other predictors using 10-fold cross-validation on the mouse dataset used in this study

Prediction	Sn	Sp	Acc	MCC
4mCPred-EL	0.8040	0.7870	0.7950	0.5910
i4mC-Mouse	0.6831	0.9020	0.7930	0.6510
4mC-CGRU	0.7763	0.8002	0.7882	0.5787
iResNetDM	0.7372	0.7815	0.7594	0.5203
TriAlignNet-4mC (ours)	0.6937	0.9125	0.8031	0.6213

Sn, sensitivity; Sp, specificity; Acc, accuracy; MCC, Matthews correlation coefficient

The independent test results (Table 2) further substantiate the model’s generalization capability. As evidenced by the tight 95% confidence intervals (CIs) derived via bootstrapping, TriAlignNet-4mC exhibited high statistical stability on unseen data. In comparative terms, our model remained highly competitive against 4mCPred-EL across most metrics, particularly in specificity, where the lower bound of our CI remained competitive against the mean performance of the baseline predictors. A critical divergence was observed in the performance trajectory of the deep learning baseline, 4mC-CGRU, which suffered a sharp decline in MCC from training to testing—a clear indicator of overfitting to local sequence motifs. Furthermore, when compared to the recent iResNetDM model, TriAlignNet-4mC demonstrated a substantial margin of improvement on unseen data, yielding an MCC of 0.6319 against iResNetDM’s 0.5065. Crucially, TriAlignNet-4mC and the feature-engineering-based i4mC-Mouse achieved closely comparable overall results (MCC of 0.6319 *vs* 0.6330, respectively) on this independent test set. In contrast to baselines that suffer from structural overfitting, TriAlignNet-4mC maintained robust performance metrics consistent with its cross-validation results. This stability confirms that explicitly modeling the DNA double-helix structure and biochemical properties acts as an effective regularization mechanism, preventing the memorization of training artifacts and ensuring reliable genome-wide applicability.

**Table 2.** Performance comparison with other predictors on the independent test set. The 95% confidence intervals (CIs) for TriAlignNet-4mC are reported in square brackets.

Model	Sn	Sp	Acc	MCC
4mCPred-EL	0.7572	0.8251	0.7910	0.5840
i4mC-Mouse	0.8071	0.8252	0.8161	0.6330
4mC-CGRU	0.7625	0.7562	0.7594	0.5188
iResNetDM	0.7375	0.7688	0.7531	0.5065
TriAlignNet-4mC (ours)	0.7937	0.8375	0.8156	0.6319
[0.7200, 0.8639]	[0.7989, 0.8744]	[0.7717, 0.8592]	[0.5506, 0.7081]

Sn, sensitivity; Sp, specificity; Acc, accuracy; MCC, Matthews correlation coefficient

### Model complexity and training stability

**Fig. 3.**
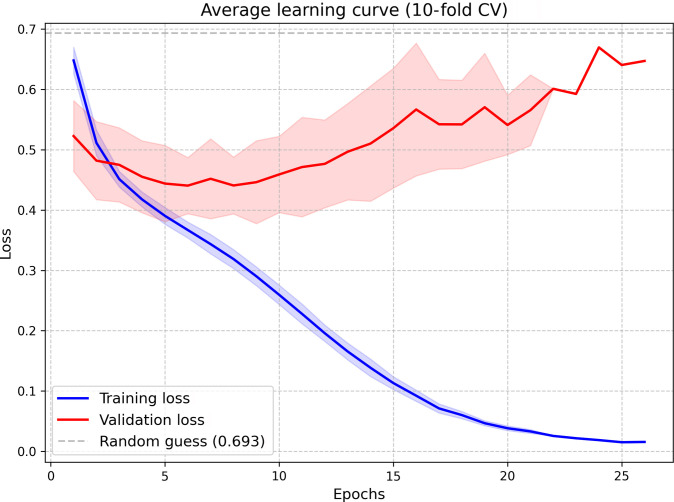
Average learning curves (loss) over 10-fold cross-validation.

To evaluate the learning capacity and convergence stability of the model, we analyzed the training dynamics across 10-fold cross-validation, as illustrated in Fig. 3. In this visualization, the blue and red curves denote the training and validation losses, respectively, with shaded regions representing the standard deviation across folds to quantify variability. The horizontal gray dashed line indicates the theoretical baseline for random guessing ($\ln\left(2\right)\approx 0.693$).

As observed, the training loss descends rapidly from the baseline toward zero, confirming that the model possesses sufficient capacity to effectively extract discriminative features from biological sequences. Concurrently, the validation loss tracks the training trajectory during the initial phase (epochs 1 to 8), reaching a distinct minimum of approximately 0.45. A characteristic divergence emerges in subsequent epochs, where the validation loss trends upward—signaling the onset of overfitting—while the training loss continues to decline. This distinct “U-shaped” trajectory empirically justifies the implementation of our early-stopping protocol. By halting training at the validation nadir (typically between epochs 8 and 12), we prevent the retention of overfitted states, thereby maximizing the generalization capability of the deployed model.

### Ablation studies

**Fig. 4.**
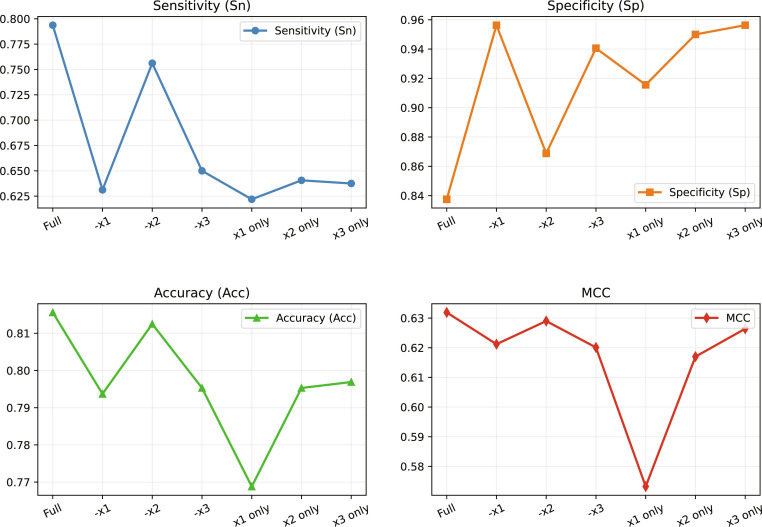
Performance comparison of ablation experiments in TriAlignNet-4mC.

To systematically quantify the contribution of individual components within TriAlignNet-4mC, we conducted a series of ablation experiments under identical training conditions, isolating specific branches to assess their distinct impact on predictive performance. The architectural variants are defined in Table 3, and the corresponding results are visualized in Fig. 4. As observed, a consistent performance degradation occurs whenever a component is excluded, validating the necessity of the full multi-branch architecture. The complete TriAlignNet-4mC model achieved the highest predictive accuracy and correlation, serving as the upper bound for performance.

**Table 3.** Summary of TriAlignNet-4mC variants in the ablation study

Model variant	Included branches	Description
Full model	x1x2x3	Complete architecture with biochemical, contextual, and structural branches
-x1	x2x3	Removes biochemical branch
-x2	x1x3	Removes contextual branch
-x3	x1x2	Removes structural branch
Only x1	x1	Uses only biochemical branch
Only x2	x2	Uses only contextual branch
Only x3	x3	Uses only structural branch

The complete TriAlignNet-4mC model achieved the highest predictive sensitivity, accuracy, and correlation, serving as the upper bound for overall performance. Specifically, the exclusion of the BioLinear branch (-x1) or the G-StructRGCN branch (-x3) precipitated the most pronounced decline in sensitivity and a noticeable reduction in overall accuracy. This demonstrates that local physicochemical properties and duplex graph topologies are indispensable for identifying true-positive 4mC sites. Interestingly, as shown in Fig. 4, while some ablated variants (such as -x1 and -x3) exhibit higher specificity, this comes at a severe detriment to sensitivity, representing a stark and imbalanced trade-off. The full TriAlignNet-4mC model effectively optimizes this trade-off, mitigating the false-negative rates to achieve the highest overall accuracy and MCC. Furthermore, the inferior performance of the single-branch baselines (such as “x1 only”) underscores the limitations of unimodal representations. In conclusion, these findings demonstrate that the 3 branches provide complementary information—biochemical, contextual, and structural—and that the position-aligned fusion mechanism plays a pivotal role in unifying these representations into a robust and balanced predictive framework.

### Model interpretability and biological sequence analysis

Beyond achieving high predictive accuracy, a critical objective of TriAlignNet-4mC is to explore the underlying computational patterns associated with benchmark 4mC candidate sites. By visualizing the learned representations from the biochemical, contextual, and structural branches, we aim to interpret how the model recognizes specific sequence and structural motifs. This analysis serves a hypothesis-generating purpose, elucidating how the model integrates local motifs, long-range dependencies, and topological constraints within the benchmark dataset.

#### Decoding long-range regulatory interactions

**Fig. 5.**
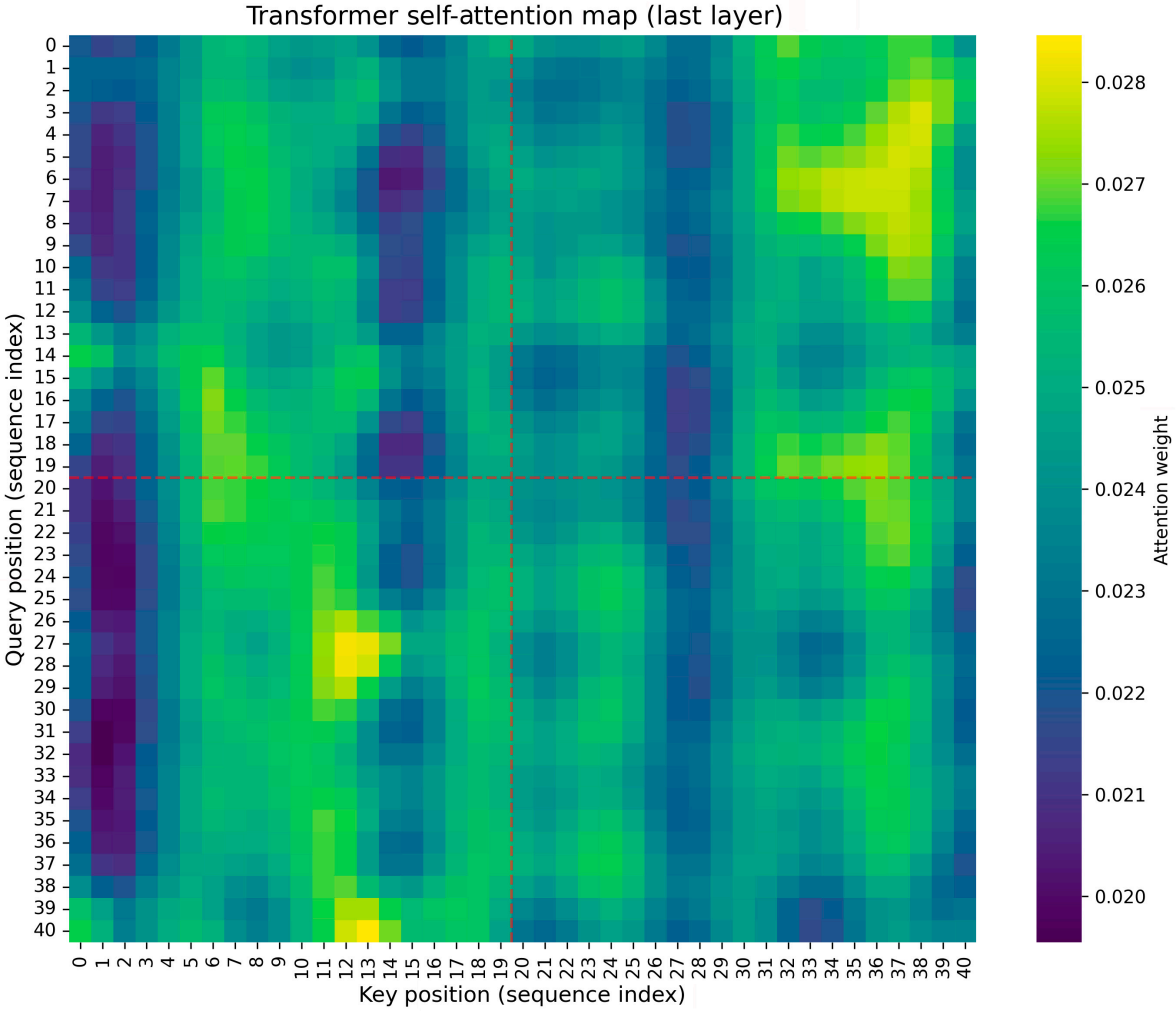
Visualization of Transformer self-attention.

The CtxFormer branch is engineered to decode global sequence semantics and infer potential regulatory interactions. To investigate the spatial dependencies captured by the model, we extracted and visualized the self-attention weights from the final Transformer layer. As illustrated in Fig. 5, the heatmap depicts the attention intensity between the query position (*y*-axis) and the key position (*x*-axis), with the intersection of the red dashed lines marking the candidate 4mC site. Notably, the high-intensity regions (indicated in yellow/green) reveal that the methylation status of the central cytosine (position 20) is not solely governed by its immediate neighbors. Instead, the model exhibits substantial attentional connectivity with distal flanking regions, specifically at indices 10 to 14 and 30 to 36. This nonlocal attention pattern suggests that TriAlignNet-4mC successfully captures long-range dependencies, potentially reflecting the functional role of DNA spatial folding or looping mechanisms that bring distal elements into physical proximity to facilitate methyltransferase recruitment.

#### Fine-grained motif discovery via IG

**Fig. 6.**
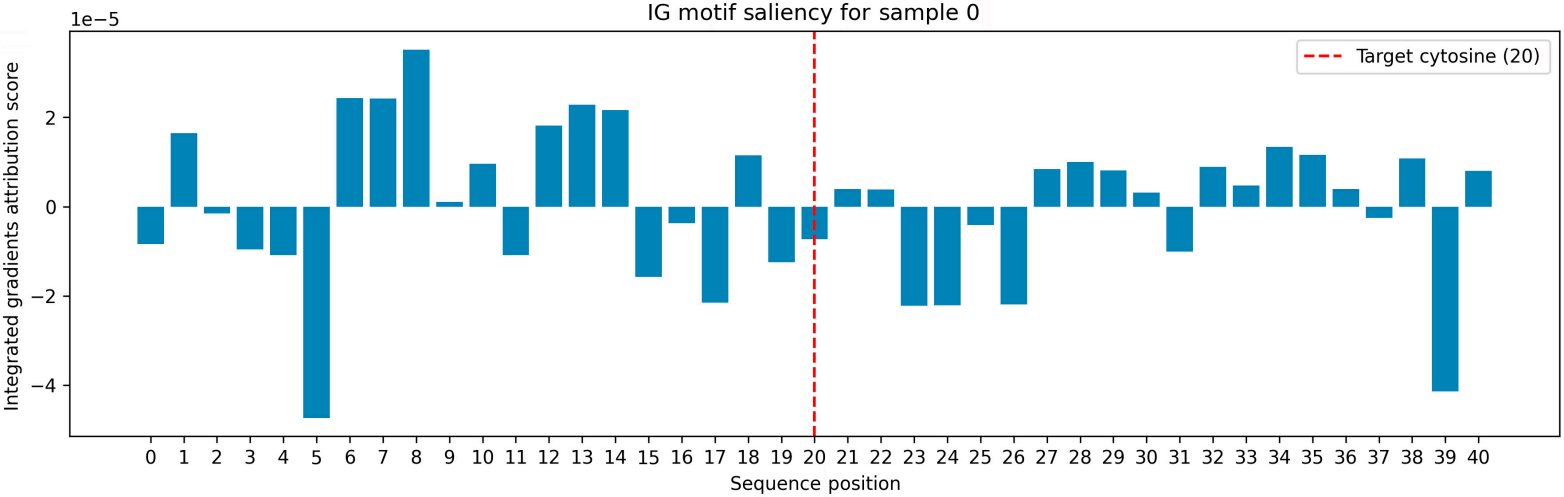
Fine-grained sequence motif attribution map via integrated gradients.

To achieve a more granular and mathematically robust understanding of the sequence features driving our model’s predictions, we upgraded our interpretability analysis by employing the axiomatic IG method. Figure 6 illustrates the fine-grained attribution profile for a representative benchmark positive sequence. Distinct from standard gradient-based saliency maps that are susceptible to local gradient saturation, the IG attribution pattern unveils a complex, bidirectional regulatory landscape within the benchmark dataset. Specifically, prominent positive attribution peaks emerge at distal upstream regions, particularly concentrated at positions 6 to 8 and 12 to 14, suggesting that the model successfully leverages the nonlocal sequence context as supportive recognition anchors. Conversely, pronounced negative attribution scores are observed at positions 5 and 39, alongside moderate suppression at positions 23, 24, and 26, indicating specific flanking loci that computationally penalize the 4mC prediction probability. These model-derived attribution patterns provide a highly structured, hypothesis-generating map of sequence dependencies. Rather than overrelying on immediate neighboring bases, TriAlignNet-4mC demonstrates an advanced capacity to weigh both collaborative and inhibitory contextual signals across the entire 41-bp window to differentiate putative methylation candidate sites.

#### Topological roles of structural features

**Fig. 7.**
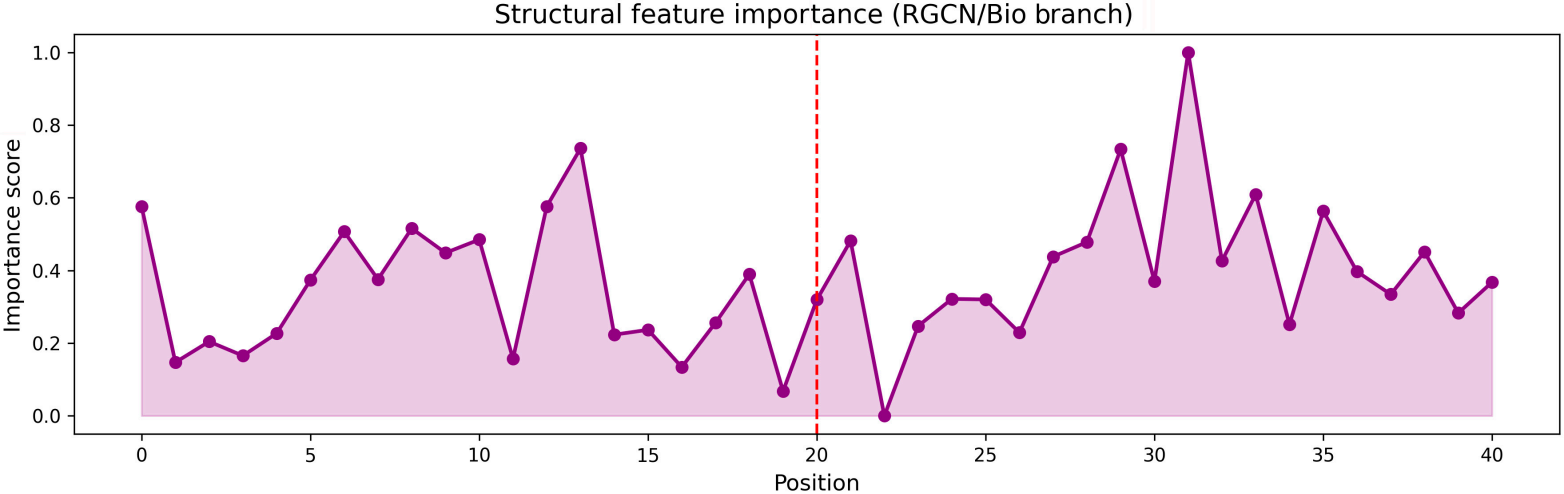
Structural feature importance distribution.

The G-StructRGCN branch interrogates the connectivity landscape of the DNA duplex to capture relational features. Figure 7 plots the structural feature importance distribution, representing the aggregated contribution of graph-derived descriptors at each position across the 41-bp window. Distinct from the localized sequence motif, the structural importance curve exhibits prominent peaks at distal positions 13 and 31, whereas the region immediately adjacent to the modification site (22 to 25) maintains a profile of lower variability. This distribution suggests that these distal loci may function as “connectivity anchors”–associated with specific information propagation constraints within the graph representation. This finding underscores the complementary role of the graph-based branch in identifying relational connectivity determinants that remain effectively invisible to purely sequence-based analyses.

### Cross-species generalization: Performance on the *D. melanogaster* benchmark

To directly address the limitations of training on smaller datasets and to rigorously evaluate the cross-species generalization capabilities of TriAlignNet-4mC, we extended our evaluation to the large-scale eukaryotic *D. melanogaster* benchmark dataset. Assessing the model on a substantially larger dataset derived from a distinct eukaryotic model organism is crucial for verifying that the proposed architecture captures fundamental, generalizable 4mC sequence patterns and relational connectivity rather than merely overfitting to murine-specific genomic distributions.

In this independent evaluation, we benchmarked TriAlignNet-4mC against classic predictors (4mCPred [37] and 4mCCNN) as well as the recently proposed advanced deep learning framework, 4mCFSNet [38]. As summarized in Table 4, TriAlignNet-4mC demonstrates an overwhelming predictive superiority across the board. Our proposed framework achieves a remarkable accuracy of 0.9012 and a high sensitivity of 0.9166. Most notably, TriAlignNet-4mC yields an exceptional MCC of 0.8029, comprehensively outperforming all existing baselines (for instance, the recent 4mCFSNet achieves an MCC of 0.7054 and an accuracy of 0.8517).

**Table 4.** Performance comparison with other predictors on the large-scale *Drosophila melanogaster* benchmark dataset

Model	Sn	Sp	Acc	MCC
4mCPred	0.8390	0.8136	0.8263	0.6500
4mCCNN	0.8640	0.8536	0.8539	0.6865
4mCFSNet	0.8898	0.8136	0.8517	0.7054
TriAlignNet-4mC (ours)	0.9166	0.8858	0.9012	0.8029

Sn, sensitivity; Sp, specificity; Acc, accuracy; MCC, Matthews correlation coefficient

The substantial performance margins observed on this large eukaryotic benchmark firmly establish the robustness, scalability, and cross-species applicability of our design. The dynamic integration of context-aware embeddings with spatial duplex-connectivity graphs enables TriAlignNet-4mC to accurately capture highly conserved epigenetic traits across diverse eukaryotic lineages, highlighting its utility as a powerful and scalable screening tool for genome-wide methylation pattern discovery.

### Validation of graph construction choices

To rigorously validate the structural assumptions inherent in the G-StructRGCN branch, we conducted comprehensive sensitivity analyses focusing on 2 critical design components: node feature dimensionality and the specific graph topology utilized for modeling DNA interactions.

#### Impact of PCA dimensionality

Given the high computational cost of processing raw BERT embeddings (768D) within a graph convolution framework, we employed PCA for dimensionality reduction. We systematically evaluated model performance across varying PCA component counts, $d\in \left\{4, 8, 16\right\}$. As detailed in Table 5, performance peaked at *d* = 8. The analysis reveals a clear trade-off: setting *d* = 4 resulted in a substantial degradation attributable to excessive information loss, whereas increasing dimensions to *d* = 16 similarly impaired performance, likely due to overfitting induced by the limited dataset size. Consequently, *d* = 8 was selected as the optimal configuration to balance feature expressiveness with computational efficiency and generalization.

**Table 5.** Sensitivity analysis on PCA dimensions

PCA dimensions	Sn	Sp	Acc	MCC
4	0.6944	0.8666	0.7847	0.5874
8 (selected)	0.7937	0.8375	0.8156	0.6319
16	0.6813	0.8724	0.7816	0.5845

PCA, principal component analysis; Sn, sensitivity; Sp, specificity; Acc, accuracy; MCC, Matthews correlation coefficient

#### Importance of double-stranded topology

Our graph construction explicitly models DNA as a double helix incorporating 3 distinct relation types: main-strand backbone continuity, complementary-strand backbone continuity, and cross-strand hydrogen-bond pairing. To empirically verify the necessity of this design, we benchmarked the full graph architecture against 2 simplified variants: (a) single-strand graph, which retains only the main-strand connectivity, and (b) no hydrogen bonds, which includes nodes from both strands but eliminates the cross-strand edges.

The results (Table 6) demonstrate that discarding complementary-strand information (single-strand) yields a notable reduction in accuracy relative to that of the proposed model. Furthermore, the full graph substantially outperformed the variant lacking hydrogen bonds. This disparity validates that the explicit modeling of base-pairing interactions is crucial for facilitating effective information flow between strands, thereby capturing the authentic topological context required to characterize 4mC sites.

**Table 6.** Validation of graph topology

Graph topology	Sn	Sp	Acc	MCC
Single-strand	0.8128	0.8018	0.8054	0.6118
No hydrogen bonds	0.6860	0.8920	0.7910	0.6012
Full graph (proposed)	0.7937	0.8375	0.8156	0.6319

Sn, sensitivity; Sp, specificity; Acc, accuracy; MCC, Matthews correlation coefficient

## Contribution and Discussion

In this study, we introduced TriAlignNet-4mC, a novel position-aligned triple-branch hybrid framework that synergistically integrates biochemical, contextual, and structural perspectives for the identification of putative DNA 4mC candidate sites. By decoupling the encoding of local physicochemical properties, long-range contextual semantics, and graph-based structural dependencies, the model fully leverages complementary information from heterogeneous modalities. A key innovation—the position-aligned late-fusion mechanism—effectively unifies these multiview features, enabling the network to learn both motif-level specifics and topology-aware representations without information loss.

Experimental evaluations via 10-fold cross-validation and independent testing on the murine benchmark demonstrate that TriAlignNet-4mC achieves highly competitive performance compared to recent advanced predictors. Rather than relying on exhaustive manual feature engineering, our framework exhibits a distinct and highly stable trade-off between sensitivity and specificity. Furthermore, extensive independent validation on the large-scale *D. melanogaster* benchmark robustly confirms the framework’s exceptional cross-species generalizability. These results highlight the model’s capacity to reliably capture the intrinsic sequence patterns and subtle structural cues associated with 4mC modification across diverse eukaryotic genomes. Rigorous ablation analyses further verified the distinct contribution of each branch, confirming that our multiview integration strategy substantially enhances generalization and acts as an effective structural regularization against overfitting.

These findings suggest that incorporating explicit graph structural modeling alongside contextual embeddings offers a promising avenue for deciphering complex methylation mechanisms. However, we explicitly acknowledge the biological simplifications inherent in our graph branch. While our duplex-connectivity graph successfully captures base-pairing and backbone relationships, it remains a discrete topological representation. It does not fully encompass fine-grained continuous DNA shape features—such as minor groove width, helix twist, or sequence deformability—nor does it account for higher-order 3D chromatin folding architectures. Integrating these continuous biophysical parameters into the spatial graph represents a vital direction for future iterations to further enhance biological realism.

In future work, we also aim to extend this framework to other epigenetic marks, such as 5mC and N6-methyladenine (6mA), to further explore cross-species transfer learning and scalability. Overall, TriAlignNet-4mC establishes a reliable and extensible computational framework for benchmark 4mC site analysis. By providing interpretable fine-grained attributions of model-derived structural anchors and long-range dependencies, TriAlignNet-4mC offers researchers an advanced candidate screening tool and generates computational hypotheses that could guide future experimental validation.

## Data Availability

The datasets and source code are available on the GitHub repository at https://github.com/hyWangFe/TriAlignNet-4mC, and the user-friendly web server is accessible at http://bio.zafuie.top/.
